# Remote ischemic preconditioning enhances aerobic performance by accelerating regional oxygenation and improving cardiac function during acute hypobaric hypoxia exposure

**DOI:** 10.3389/fphys.2022.950086

**Published:** 2022-09-09

**Authors:** Zhifeng Zhong, Huaping Dong, Yu Wu, Simin Zhou, Hong Li, Pei Huang, Huaijun Tian, Xiaoxu Li, Heng Xiao, Tian Yang, Kun Xiong, Gang Zhang, Zhongwei Tang, Yaling Li, Xueying Fan, Chao Yuan, Jiaolin Ning, Yue Li, Jiaxin Xie, Peng Li

**Affiliations:** ^1^ Department of High Altitude Operational Medicine, College of High Altitude Military Medicine, Army Medical University (Third Military Medical University), Chongqing, China; ^2^ Department of Anesthesiology, Xinqiao Hospital, Army Medical University (Third Military Medical University), Chongqing, China; ^3^ Key Laboratory of High Altitude Medicine, PLA, Army Medical University (Third Military Medical University), Chongqing, China; ^4^ Key Laboratory of Extreme Environmental Medicine, Ministry of Education of China, Army Medical University (Third Military Medical University), Chongqing, China; ^5^ Department of Anesthesiology, First Affiliated Hospital, Army Medical University (Third Military Medical University), Chongqing, China

**Keywords:** hypobaric hypoxia, remote ischemic preconditioning, maximal oxygen uptake, regional oxygenation, cardiac function, thymosin beta-4, heat shock proteins

## Abstract

Remote ischemic preconditioning (RIPC) may improve exercise performance. However, the influence of RIPC on aerobic performance and underlying physiological mechanisms during hypobaric hypoxia (HH) exposure remains relatively uncertain. Here, we systematically evaluated the potential performance benefits and underlying mechanisms of RIPC during HH exposure. Seventy-nine healthy participants were randomly assigned to receive sham intervention or RIPC (4 × 5 min occlusion 180 mm Hg/reperfusion 0 mm Hg, bilaterally on the upper arms) for 8 consecutive days in phases 1 (24 participants) and phase 2 (55 participants). In the phases 1, we measured the change in maximal oxygen uptake capacity (VO_2_max) and muscle oxygenation (SmO_2_) on the leg during a graded exercise test. We also measured regional cerebral oxygenation (rSO_2_) on the forehead. These measures and physiological variables, such as cardiovascular hemodynamic parameters and heart rate variability index, were used to evaluate the intervention effect of RIPC on the changes in bodily functions caused by HH exposure. In the phase 2, plasma protein mass spectrometry was then performed after RIPC intervention, and the results were further evaluated using ELISA tests to assess possible mechanisms. The results suggested that RIPC intervention improved VO_2_max (11.29%) and accelerated both the maximum (18.13%) and minimum (53%) values of SmO_2_ and rSO_2_ (6.88%) compared to sham intervention in hypobaric hypoxia exposure. Cardiovascular hemodynamic parameters (SV, SVRI, PPV% and SpMet%) and the heart rate variability index (Mean RR, Mean HR, RMSSD, pNN50, Lfnu, Hfnu, SD1, SD2/SD1, ApEn, SampEn, DFA1and DFA2) were evaluated. Protein sequence analysis showed 42 unregulated and six downregulated proteins in the plasma of the RIPC group compared to the sham group after HH exposure. Three proteins, thymosin β4 (Tβ4), heat shock protein-70 (HSP70), and heat shock protein-90 (HSP90), were significantly altered in the plasma of the RIPC group before and after HH exposure. Our data demonstrated that in acute HH exposure, RIPC mitigates the decline in VO_2_max and regional oxygenation, as well as physiological variables, such as cardiovascular hemodynamic parameters and the heart rate variability index, by influencing plasma Tβ4, HSP70, and HSP90. These data suggest that RIPC may be beneficial for acute HH exposure.

## 1 Introduction

Exposed to an altitude of 2,500 m or above, the human body will experience physiological changes such as an increase in heart rate and respiratory rate and a decline in blood oxygen saturation, and some unacclimatized persons may also suffer from acute mountain sickness due to hypoxia (Leissner and Mahmood, 2009; Roach et al., 2018). A gradual decrease in atmospheric pressure occurs with increasing elevation, resulting in hypobaric hypoxia (HH) (Moon et al., 2016a; Moon et al., 2016b; Park et al., 2022b). The decrease in the partial pressure of oxygen (O_2_) will finally lead to a reduction in O_2_ availability. The reduction in O_2_ availability threatens aerobic metabolism, and maintenance of an acceptable aerobic scope for activity is a major challenge (Calbet et al., 2016; McClelland and Scott, 2019). The decrease in aerobic performance during HH exposure and the negative impact of altitude on aerobic performance capacity are due to a decreased availability of O_2_ in the pulmonary alveoli or muscle microvasculature (Chapman et al., 2013; Calbet et al., 2016). Acclimatization may compensate for low availability of O_2_ by adjusting physiological delivery and improving aerobic performance (Calbet et al., 2003). Hence, it is necessary to develop time- and cost-efficient preacclimatization and/or conditioning strategies to facilitate and/or accelerate high-altitude acclimatization for various performance purposes.

Enhancing O_2_ delivery helps attenuate the detrimental impact of altitude. As a result, much research has been devoted to balancing the effects of hypoxia before or when reaching a plateau (Buchheit et al., 2013; McClelland and Scott, 2019; Storz and Scott, 2019). These strategies include hypoxia-based preacclimatization strategies, including artificial hypobaric chamber exposure, intermittent hypoxic training, and progressive ascent to plateau (Millet et al., 2010; Chapman et al., 2013; Lu et al., 2020). However, the associated time and financial costs make these strategies difficult or impossible to implement for some people, such as military personnels or travelers with limited time.

Remote ischemic preconditioning (RIPC) is a convenient strategy for intermittent occlusion and reperfusion of peripheral blood flow to protect against subsequent events of similar or greater ischemia and hypoxia stress (Murry et al., 1986; Ekeloef et al., 2019; Pearce et al., 2021). This method can increase the delivery of O_2_ (Moses et al., 2005) and promote a higher muscle O_2_ utilization (Paradis-Deschenes et al., 2016) with an altered deoxygenation to enhance performance (Barbosa et al., 2015; Kido et al., 2015; Tanaka et al., 2016; Richard and Billaut, 2018; Caru et al., 2019). Moreover, RIPC reduces blood pressure (Pryds et al., 2017), improves ventricular function, and cardiac output (Honda et al., 2019) to elicit cardioprotection. What’s more, the protection may come from plasma-derived humoral mediation (Michelsen et al., 2012; Heusch et al., 2015). In addition, RIPC has the potential to facilitate high-altitude acclimatization with an amelioration of aerobic performance (Paradis-Deschenes et al., 2018; da Mota et al., 2019). However, there have been very few studies about RIPC’s effect on aerobic performance at an altitude of approximately 4,000 m or above (Hittinger et al., 2015; Paradis-Deschenes et al., 2018; Caru et al., 2019; da Mota et al., 2019; Wiggins et al., 2019), and the physiological variables and underlying mechanisms remain unclear.

In this study, we investigated the effects of RIPC on aerobic performance compared to a sham group. We monitored the maximal oxygen uptake capacity (VO_2_max) through a graded exercise test and muscle oxygenation (SmO_2_) was also measured meanwhile, recorded cardiovascular hemodynamic parameters and the heart rate variability index during HH exposure, and explored the underlying mechanisms. We hope that our findings will broaden the current knowledge regarding the mechanism of the protective effect of RIPC. We hypothesize that RIPC is a more easily implemented, cost-effective training strategy that can be performed to facilitate and/or accelerate high-altitude acclimatization in large groups.

## 2 Materials and methods

### 2.1 Participants and study design

Seventy-nine adult males participated in the study. All participants had a healthy body and completed the medical questionnaire before testing. The exclusion criteria included 1) having acute or chronic illness (including generalized anxiety disorder, depression, cardiovascular, respiratory, movement and metabolic diseases), 2) having taken medications within the previous 3 months, 3) having a drinking or smoking habit, 4) having an experience with high altitude (>2,500 m), 5) having been enrolled in another clinical test within 3 months. All participants provided written informed consent after being informed of the experimental procedures, potential benefits and risks. The study was approved by the Medical Ethics Committee of the Army Medical University (Ethical serial number: 2020-003-03) and was conducted in accordance with the ethical principles of the Declaration of Helsinki. Written informed consent was obtained from all participants.

**FIGURE 1:**
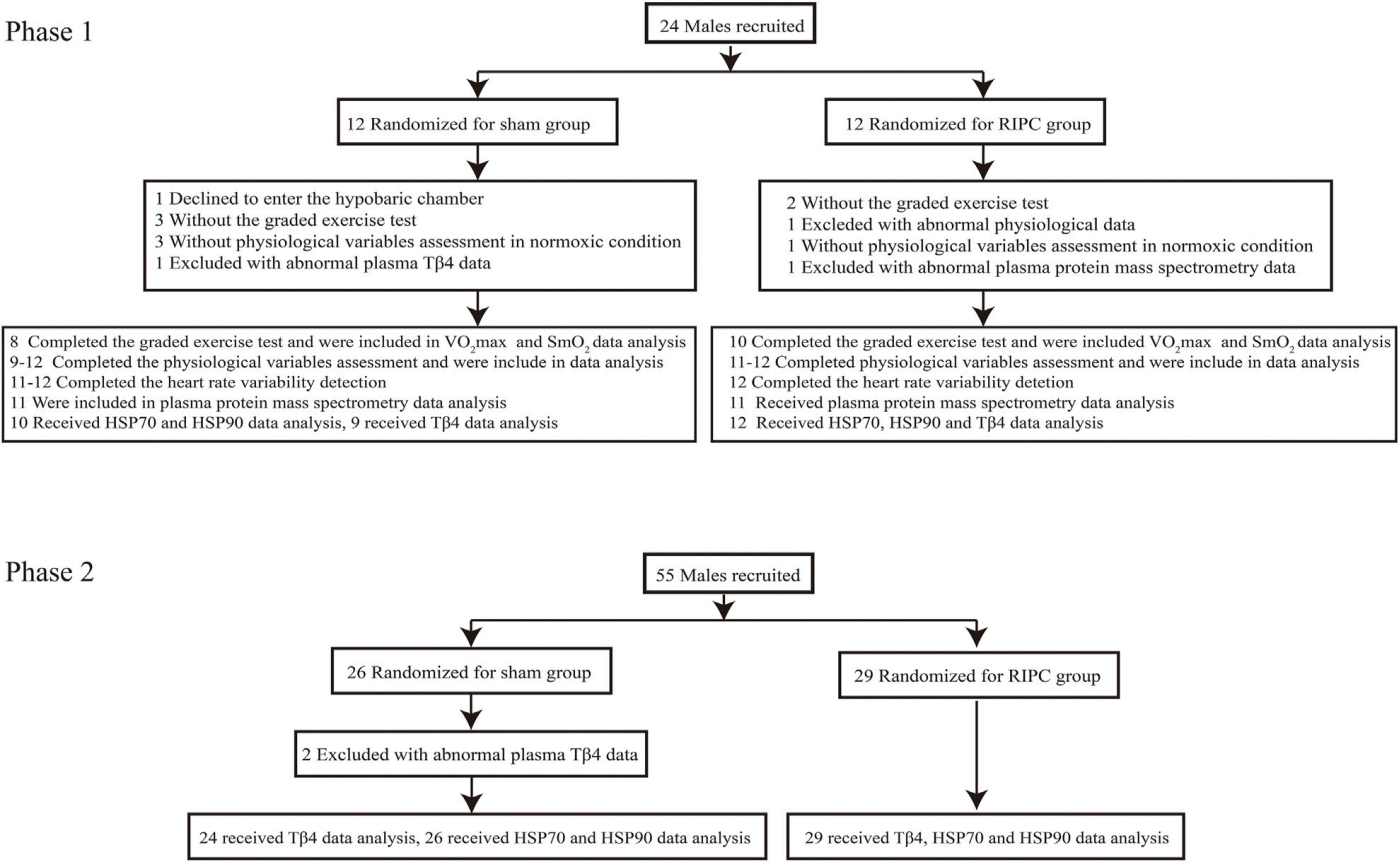
The recruitment flow diagram.

The 79 participants were divided into two phases, phase 1 (24 participants) and phase 2 (55 participants), based on their own choice. Each phase was randomly divided into two groups, the sham group and RIPC group. The anthropometric data is shown in Table 1 and the recruitment flow diagram is shown in Figure 1.

**TABLE 1:** Characteristics of participants in the sham and RIPC groups.

Characteristics	Sham Group	RIPC Group
Phase 1		
Group size/sex	12 males	12 males
Age (years)	24.17 ± 1.99	23.75 ± 1.29
Weight (kg)	65.33 ± 5.18	67.42 ± 7.51
Height (cm)	171.33 ± 4.54	171.92 ± 4.19
BMI(kg/m^2^)	22.23 ± 1.10	22.80 ± 2.33
Phase 2		
Group size/sex	26 males	29 males
Age (years)	20.38 ± 1.70	20.17 ± 1.58
Weight (kg)	67.35 ± 7.82	65.65 ± 8.61
Height (cm)	173.35 ± 5.58	171.97 ± 6.58
BMI(kg/m^2^)	22.36 ± 1.92	22.13 ± 2.02

Data are presented as mean ± standard error of the mean. RIPC, remote ischemic preconditioning, BMI: body mass index.

**FIGURE 2:**
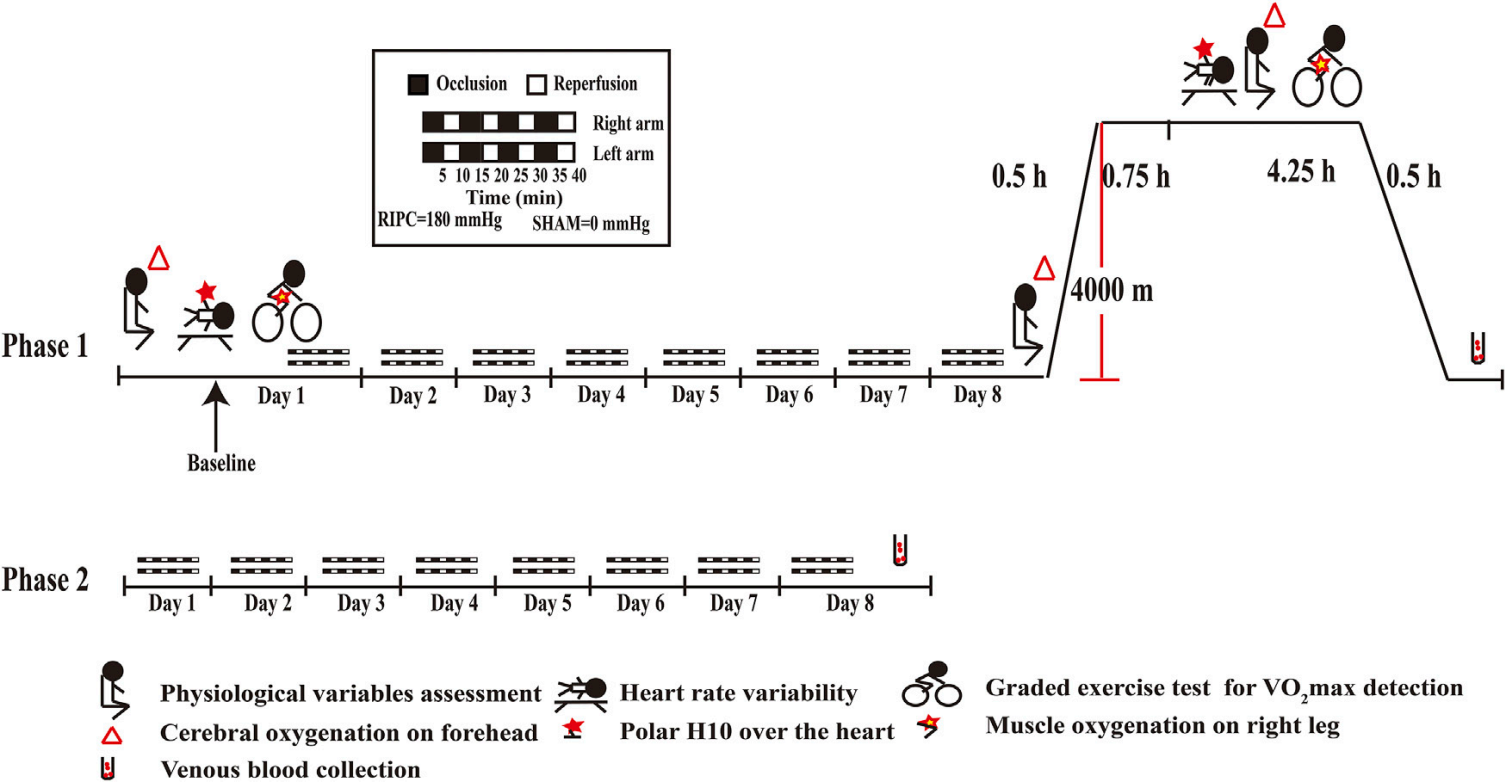
Schematic diagrams of RIPC intervention and examinations. RIPC: remote ischemic preconditioning.

For the phase 1 (As shown in Figure 2), it was carried out at a low altitude (approximately 400 m, Chongqing, China) and in a hypobaric chamber (462 mmHg, 4,000 m, 22 ± 5°C, 40–45% relative humidity). On the first day, physiological variables and heart rate variability index were measured after stabilization. Then, VO_2_max and SmO_2_ were measured when they were taking a graded exercise test. Thereafter, the RIPC intervention, an effective method to prevent acute mountain sickness (Wang et al., 2021) and enhance sports performance (Lisboa et al., 2017; Richard and Billaut, 2018), began and was performed continued for 8 days. On the eighth day, after the last RIPC intervention, physiological variables were measured under normoxia and then the 24 participants underwent a 30-min ascent profile from the 400–4,000 m in a hypobaric chamber. After sufficient rest (45 min), physiological variables, heart rate variability index, VO_2_max and SmO_2_ were measured, it lasted for 4.25 h. A peripheral venous blood sample was collected after they returned to normoxia condition.

For the phase 2 (As shown in Figure 2), it was carried out at a low altitude (approximately 400 m, Chongqing, China). The 55 participants performed the RIPC intervention continued for 8 days and provided peripheral venous blood samples on the eighth day.

### 2.2 RIPC protocol

RIPC was performed by placing a blood pressure cuff (Huayingtai Medical Co., Ltd., Shenzhen, China) around the left and right upper arms, inflating it to a pressure of approximately 180 mmHg for 5 min followed by deflation for 5 min, as described in previous reports (Lisboa et al., 2017; Richard and Billaut, 2018). The pressure applied in the sham group was 0 mmHg. This protocol was repeated for four cycles, which took 40 min [4 ×(5min of arterial occlusion +5min of non−occlusion)=40 min ]. RIPC was carried out daily for 8 days at a low altitude. A schematic diagram is shown in Figure 2.

### 2.3 Baseline Physiological Variable Assessment

Baseline physiological variables were measured using a Masimo SET Radical-7 oximeter (Masimo Corporation, Irvine, CA, United States) and a CNAP monitor 500 (CNSystems Medizintechnik AG, Graz, Austria). Participants were seated and asked to rest for 5 min at the beginning of the procedure. After cleaning the skin and nails, a CNAP double-finger cuff was placed on the arm with a pressure transducer on the forearm and an oscillometric cuff on the upper arm for calibration. SBP (mmHg), DBP (mmHg), mean arterial pressure (MAP, mmHg), pulse (bpm), pulse rate (PR, bpm), stroke volume (SV, ml), stroke volume index (SI, ml/m^2^), systemic vascular resistance (SVR, dyn*s/cm^5^), systemic vascular resistance index (SVRI, dyn*s/cm^5^), pulse pressure variation (PPV%), cardiac output (CO, l/min), and cardiac index (CI, l/min/m^2^) were recorded using the CNAP. The Masimo pulse oximeter probe was placed on the index finger, and the Masimo O3™ near-infrared spectroscopy (NIRS) monitor connected to a regional oximetry system was placed on the participant’s forehead, as previously described (Redford et al., 2014; Lee et al., 2022). Patient state index (PSI), peripheral oxygen saturation (SpO_2_), cerebral oximetry (rSO_2_), PR (bpm), methemoglobin saturation (SpMet), Pleth variability index (PVi), and peripheral perfusion index (PI) were recorded using the Masimo SET Radical-7 oximater. Cerebral fractional tissue oxygen extraction (cFTOE) [cFTOE = (SpO_2_ − rSO_2_)/SpO_2_] was calculated to reflect the balance between oxygen delivery and oxygen consumption. The baseline physiological variables were recorded when stable data were displayed.

### 2.4 Heart rate variability detection

All participants were informed to refrain from moderate-to-heavy physical activity and consumption of caffeinated or alcoholic beverages 12 h before the monitoring procedure. Each participant rested for 5 min prior to the procedure to ensure the stabilization of heart rate (HR), and the participants were informed that they could not talk, move, or sleep during the procedure. RR intervals (RRi) were recorded between 09:30 A.M and midday, with the participant in a supine position, using a heart rate monitor (Polar H10; Polar Electro Oy, Kempele, Finland), which was connected to a smartphone app SelfLoops HRV (SelfLoops, Fermo, Italy) via Bluetooth. Continuous RRi was recorded for at least 5 min after the HR stabilized. The Kubios HRV standard (ver. 3.3.1; Kubios Oy) was used to analyze the RRi data. The Kubios HRV standard contains several filter levels in which the identified artifacts are automatically replaced with interpolated values using cubic spline interpolation. The Kubios filter in our analysis was set at a “low” level. In the present study, HRV indices commonly used in the time, frequency, and non-linear domains were used. The indices adopted in the time-domain analysis were mean RRi, standard deviation of NN intervals (SDNN), mean HR, root mean square of successive differences (RMSSD), number of pairs of successive NNs that differ by more than 50 ms (NN50), proportion of NN50 divided by the total number of NN (R-R) intervals (pNN50), RR triangular index (RRTI), triangular interpolation of NN intervals (TINN). The power spectra of the components were calculated using the fast Fourier transform in frequency-domain analysis. The indices adopted in the frequency-domain analysis were very low frequency (VLF, ≤0.04 Hz), low frequency (LF, 0.04–0.15 Hz), high frequency (HF, 0.15–0.4 Hz), LF/HF ratio, LF and HF expressed in normalized units, and total power (TP). The indices adopted in the nonlinear domain analysis were the standard deviation of the Poincaré plot perpendicular to (SD1) and along (SD2) the line-of-identity, SD2/SD1, approximate entropy (ApEn), sample entropy (SampEn), and detrended fluctuation analysis: short-term (DFA1) and long-term (DFA2) fluctuation slope.

### 2.5 Maximal oxygen uptake (VO_2_max)

A brake bicycle ergometer (Cosmed, Rome, Italy) and a Fitmate GS calorimeter (Labiopro, Kyiv, Ukraine) were used for the study. Participants were instructed to arrive at the laboratory after fasting for ≥ 2 h and be well rested and hydrated. Participants performed 5 min of unloaded pedaling as a warm-up, followed by a graded exercise test. Because volunteers often participate in physical exercise and have a relatively good physical quality, we chose the intensity started with 0 W and increased by 30 W every minute as previously described (Wilcox et al., 2016). The cycling frequency was maintained between 50 and 60 revolutions per minute (rpm) throughout the exercise. Oxygen uptake was continuously measured using open-circuit spirometry (True-One 2,400; Parvo Medics, Sandy, UT, United States). HR was continuously recorded using a HR monitor (Polar H10; Polar Electro Inc., Lake Success, NY, United States). In this study, oxygen uptake was considered maximum (Park et al., 2022a) when the following two criteria were satisfied: 1) there was no further increase in oxygen uptake despite a further increase in intensity, 2) when the heart rate was age-predicted HRmax (85%* (220 – age in years)). The value of VO_2_max was calculated according to the instrument’s own algorithm.

### 2.6 Muscle oxygenation

The Moxy Monitor (Fortiori Design LLC, Hutchinson, MN, United States) is a continuous-wave near-infrared spectroscopy device that provides validity of muscle oxygen saturation (SmO_2_) measurements on a scale of 0–100% (Feldmann et al., 2019). After rubbing and cleaning the skin with alcohol swabs, the optodes were attached to the skin on the lower third of the quadriceps femoris on the right thigh. The monitors have four light-emitting diodes with wavelengths of 680 nm, 720 nm, 760 nm, and 800 nm. The device detectors were spaced between 1.25 and 2.5 cm from the emitter. The default sampling rate cycle through the four wavelengths was 80 times every 2 s, and the average output rate for reading was 0.5 Hz. Relative changes in SmO_2_ were determined on the basis of the modified Beer–Lambert law during the graded exercise test. All SmO_2_ values were recorded every second when the participants performed the graded exercise test.

### 2.7 Peripheral venous blood collection

Peripheral venous blood was collected into EDTA-coated tubes by the qualified nurses from a hospital after HH exposure when the altitude returned to the sea level (Phase 1) and before HH exposure (Phase 2). After collection, the blood samples were centrifuged at 2000 × g for 10 min at 4 C. Plasma was extracted and stored at −80 C for later use.

### 2.8 Plasma protein mass spectrometry (MS) acquisition and analysis

The Shanghai Applied Protein Technology Co., Ltd. provided plasma proteomics services. Plasma sample preparation and digestion were performed as previously described (Fang et al., 2020). Equal aliquots from each sample were pooled into one sample for data dependent acquisition (DDA) library generation. The pooled sample was analyzed using a Q Exactive HF X mass spectrometer (Thermo Scientific, Waltham, MA, United States) connected to an Easy nLC 1,200 chromatography system (Thermo Scientific). The peptides from each sample were analyzed using liquid chromatography (LC)-MS)/MS in data-independent acquisition (DIA) mode. Each DIA cycle included one complete MS-selected ion monitoring (SIM) scan and 30 DIA scans covered a mass range of 350–1800 m/z. The parameters of the DIA mode scan were set as follows. The full resolution of the SIM full scan was 120,000 at 200 m/z; automatic gain sham (AGC), 3e6; maximum ion trap (IT) time, 50 ms; profile mode, the DIA scans were set at a resolution of 15,000; AGC target, 3e6; Max IT, auto; normalized collision energy, 30 eV. The runtime cost was 120 min, with a linear gradient of buffer B (0.1% formic acid and 84% acetonitrile) at a flow rate of 250 nl/min. The quality control sample (the aforementioned pooled sample) was injected in the DIA mode at the beginning of the MS experiment and after every six injections to monitor the MS performance. For the DDA library data, spectronaut TM 14.4.200727.47784 software (Biognosys, Schlieren, Switzerland) was used to search for the FASTA sequence of the DDA library data, and the Swiss_Prot database was used. For the DIA data, qualitative and quantitative analysis were carried out by searching the constructed spectral library.

### 2.9 Validation by ELISA test

Plasma samples were thoroughly thawed and mixed, and concentrations of thymosin β4 (Tβ4), heat shock protein-70 (HSP70), and heat shock protein-90 (HSP90) were tested using the corresponding ELISA kits (Cusabio Biotech Co., Ltd., Wuhan, China). All assays were performed using the recommended buffers, substrates, and diluents. Following the manufacturer’s instructions, after incubation with the 3.3′5.5′ tetramethylbenzidine (TMB) substrate solution, the reaction was terminated by adding the stop solution. The absorbance was determined at 450 nm, and the concentrations were calculated according to the standard curve.

### 2.10 Statistical Analysis

Sample size was calculated using PASS (version PASS 2021; NCSS LLC, Kaysville, UT, United States) and taking into account a two-sample *t* test power analysis. Using the group mean and standard deviation (SD) of previous studies which have successfully shown an effect of the VO_2_max (Elisabeth et al., 2017) and HSP70 (Yan et al., 2020) following pharmacological intervention, the software suggests that 9 participants for each group in phase 1 and 12 participants for each group in phase 2 were enough to achieve a two-sided significance level of 0.05, with 0.80 of power.

All data were analyzed using SPSS (version 16.0; SPSS Inc., Chicago, IL, United States) and are presented as mean ± standard error or standard deviation of the mean (SEM or SD). Based on the data distribution using the Shapiro–Wilk’s test, independent *t*-test was used to analyze normal distribution data (e.g., VO_2_max between sham group and RIPC group in HH exposure, Tβ4, HSP70, and HSP90), nonparametric test followed by Mann–Whitney *U* test was used to analyze abnormal distribution (e.g., maximum of SmO_2_ between normoxic condition and HH condition, VO_2_max between normoxic condition and HH condition in sham group). Repeated measures two-way analysis of variance (ANOVA) with time as the repeated measure factor and the intervention as the between-group factor was used to analyze SBP, DBP, MAP, Pulse, PR, SV et al.,. As the Mauchly’s test showed that sphericity was violated, the degrees of freedom were corrected to adjust ANOVA results using Greenhouse–Geisser estimates of sphericity. *p* < 0.05 was considered to indicate statistical significance.

For quality control of the proteomic data, the protein with a quantitative value in at least 50% of samples within a group were used for subsequent analysis according to the general principle. The proteins were significantly upregulated or downregulated if the fold change of the RIPC group/sham group was >1.5 or <0.67, respectively, and the *p*-value was <0.05 (as determined by a *t*-test). A hierarchical clustering analysis was performed to group the differentially expressed proteins in the two groups. The differentially expressed proteins were then searched in the Gene Oncology (GO) and Kyoto Encyclopedia of Genes and Genomes (KEGG) databases to determine the important biological processes and key signaling pathways involved in the protective effect of RIPC by using Clusterprofile 4.0 R package. The simplify function in clusterProfiler 4.0 was used to remove redundancy from the GO enrichment results. The terms or pathways with an adjusted *p* value (*p*.adjust) less than 0.05 were considered to be significant.

## 3 Results

### 3.1 RIPC improves VO_2_ max

**FIGURE 3:**
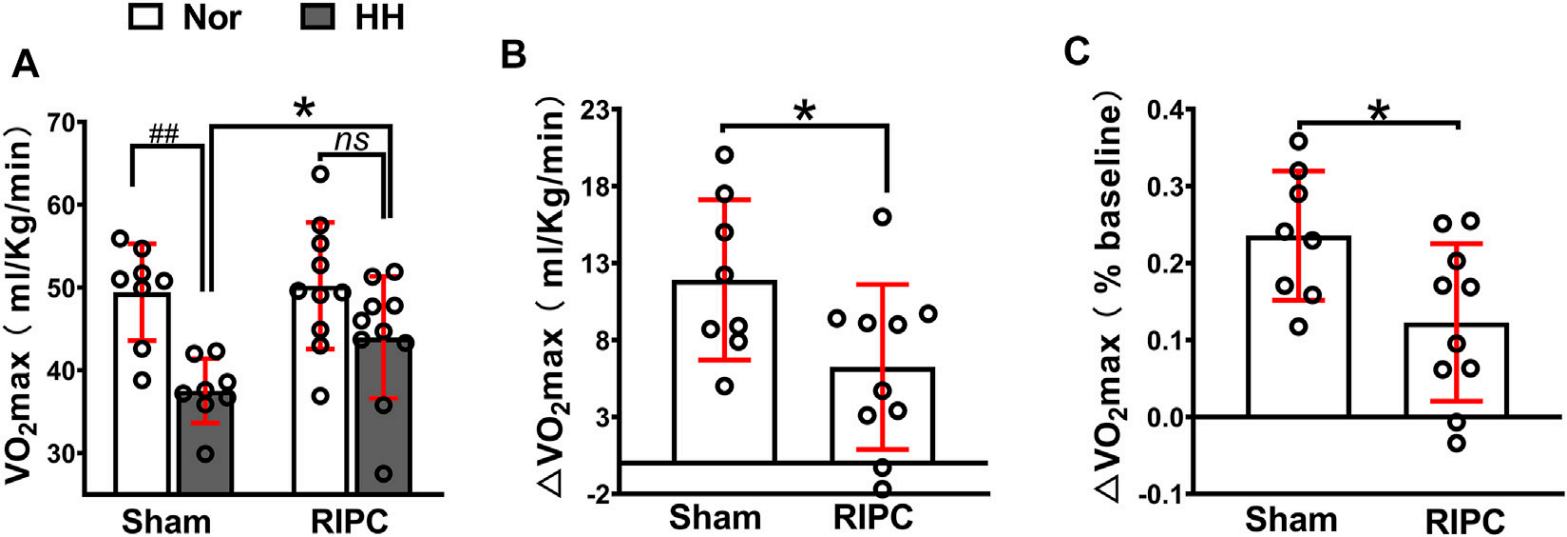
Variation of VO_2_max in the sham and RIPC groups under normoxic and HH conditions. **(A)** VO_2_ max measurement **(B)** ∆VO_2_max and**(C)** percent changes of VO_2_max from the normoxic to HH condition in each group. Values are presented as mean ± standard deviation. ^###^ *p* < 0.01 vs. normoxic; *p < 0.05, *p < 0.001 vs. sham group. RIPC: remote ischemic preconditioning, Nor: normoxic; HH: hypobaric hypoxia, VO_2_ max: maximal oxygen uptake capacity.

A graded exercise test evaluated VO_2_max to identify whether RIPC could improve aerobic performance during the subsequent HH exposure (Figure 3). Consistently, our data showed that acute HH exposure caused a decrease in VO_2_max (normoxic: 49.43 ± 5.84 ml kg^−1^ min^−1^ and hypobaric hypoxia: 37.52 ± 3.88 ml kg^−1^ min^−1^; *p <* 0.01, Figure 3A) during a graded exercise test (Fulco et al., 1998; Wehrlin and Hallen, 2006), but the VO_2_max in the RIPC group showed no difference under normoxic and HH conditions (normoxic: 50.21 ± 7.65 ml kg^−1^ min^−1^ and hypobaric hypoxia: 43.97 ± 7.37 ml kg^−1^ min^−1^; *p >* 0.05, Figure 3A). Moreover, VO_2_max under the HH condition showed a difference between the sham and RIPC groups (sham group: 37.52 ± 3.88 ml kg^−1^ min^−1^ and RIPC group: 43.97 ± 7.37 ml kg^−1^ min^−1^; *p* < 0.05, Figure 3A), indicating that RIPC intervention could alleviate the HH-induced decrease in aerobic performance. Meanwhile, ∆VO_2_ max showed less change after RIPC (sham group: 11.91 ± 5.21 ml kg^−1^ min^−1^ and RIPC group: 6.24 ± 5.37 ml kg^−1^ min^−1^; *p* < 0.05, Figure 3B). Furthermore, from normoxic to HH conditions, the mean percent change in VO_2_ max (∆VO_2_ max%) was reduced by approximately 11.29% and was statistically different (sham group: 24.00 ± 8.40% and RIPC group: 12.27 ± 10.23%; *p* < 0.01, Figure 3C) between the two groups. An increase in VO_2_ max is important because it indicates an increase in the long-term health status and exercise tolerance (Myers et al., 2002). Despite the widely observed increase in VO_2_max was demonstrated in response to RIPC, the specific adaptations explaining this outcome remain elusive.

### 3.2 RIPC ameliorates regional oxygenation

**FIGURE 4:**
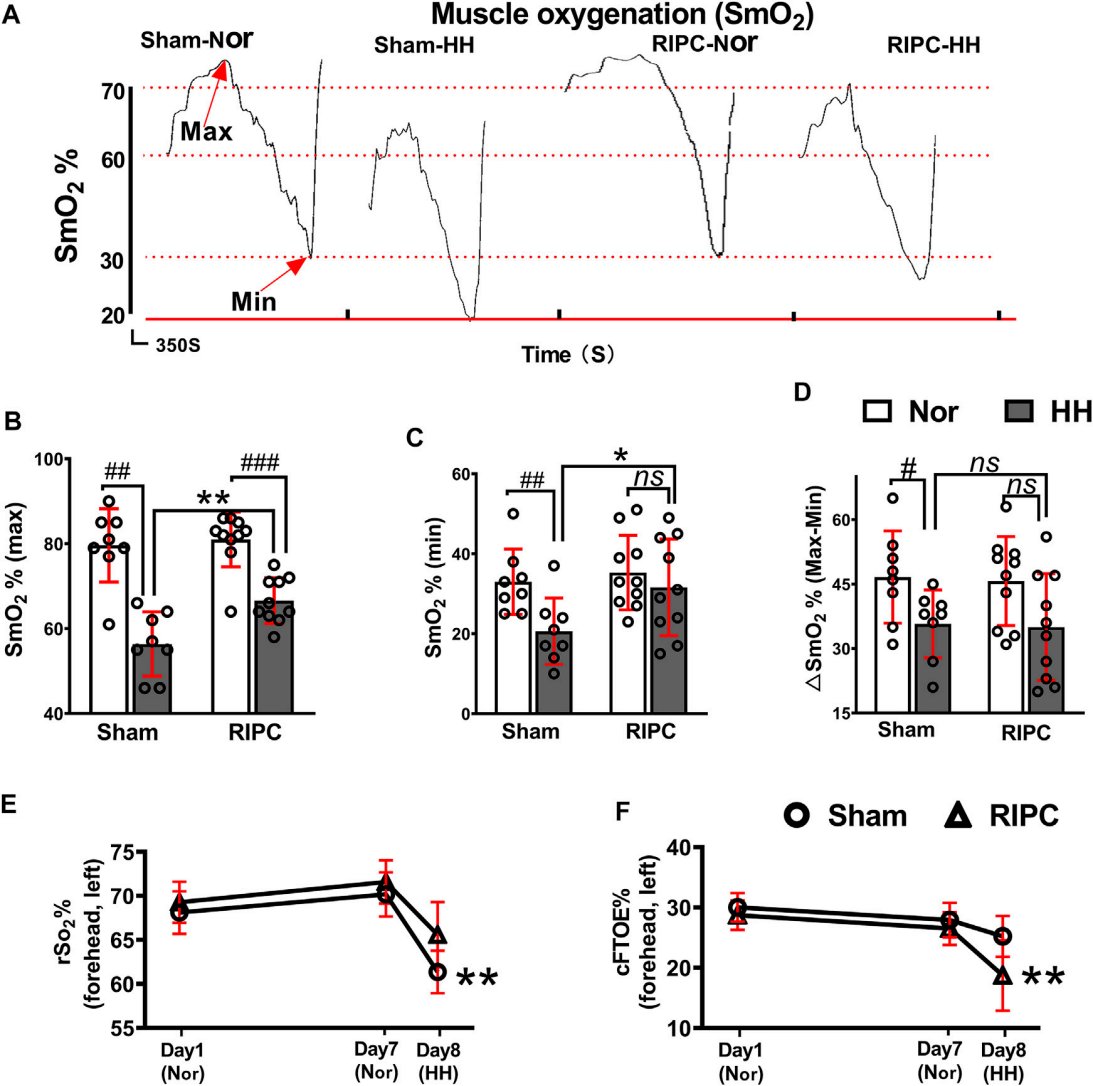
The beneficial effects of RIPC on SmO_2_ and rSO_2_. **(A)** SmO_2_ results from representative participants under different environments. **(B)** The maximum, **(C)** minimum, and **(D)** ∆SmO_2_ from the normoxic to HH condition in each group. ^#^ *p* < 0.05, ^##^ *p* < 0.01, ^###^ *p* < 0 .001 vs. normoxic; *p < 0.05 and **p < 0.01vs. sham group. **(E)** Variation of rSO_2_% and **(F)** cFTOE% in the sham and RIPC groups at day 1, day 7, and day 8 in the left brain. **p < 0.01 vs. sham group. Values are presented as mean ± standard deviation. Nor: normoxic, HH: hypobaric hypoxia, SmO_2_: muscle oxygenation, rSO_2_: regional cerebral oxygenation, cFTOE: cerebral fractional tissue oxygen extraction.

SmO_2_ change can be used to evaluate oxygenation capacity and is correlated with exercise performance (Perrey and Ferrari, 2018; Feldmann et al., 2020). In the graded exercise test, as exercise intensity increased, there was a transient increase in SmO_2_, followed by a subsequent decline (Figure 4A). In the normoxic condition, there were no differences in any variables between the sham and RIPC groups (Figures 4B–D). Consistently, the absolute maximum (normoxic: 79.63 ± 8.63% and hypobaric hypoxia: 56.38 ± 7.58% for SmO_2_; *p <* 0.01, Figure 4A) and minimum (normoxic: 33.00 ± 8.18% and hypobaric hypoxia: 20.63 ± 8.30% for SmO_2_; *p <* 0.01, Figure 4B)of SmO_2_ and ∆SmO_2_ (normoxic: 46.63 ± 10.73% and hypobaric hypoxia: 35.75 ± 7.89% for ∆SmO_2_; *p <* 0.05, Figure 4C) were reduced under the HH condition in the sham group (Calbet et al., 2009), whilst there were no statistically significant changes in the absolute minimum of SmO_2_ and ∆SmO_2_ between the HH and normoxia condition in the RIPC group (Figures 4C,D). Notably, the absolute maximum (sham group: 56.38 ± 7.58% and RIPC group: 66.60 ± 5.38% for SmO_2;_ *p <* 0.01, Figure 4A) and minimum (sham group: 20.63 ± 8.30% and RIPC group: 31.60 ± 12.10% for SmO_2;_ *p <* 0.05, Figure 4B) of SmO_2_ were significantly different between the two groups under the HH conditions, and RIPC intervention accelerated both the maximum (18.13%) and minimum (53%). Overall, rSO_2_ can be considered a meta-parameter influenced by ventilation, oxygenation, hemoglobin (Hb) brain perfusion, and metabolism. The normal range of rSO_2_ suggests a balance between O_2_ supply and consumption in the individual regions of the brain (Scheeren, 2016; Ferraris et al., 2018). In the present study, a marked change in the peripheral rSO_2_ value of the left forehead was observed. In particular, a significant increase in rSO_2_ value was found in the left forehead after RIPC compared to that in the sham group in the HH condition (sham group: 61.36 ± 2.42% and RIPC group: 65.58 ± 3.70%; *p* < 0.01, Figure 4E). Meanwhile, the cFTOE, which is calculated to reflect the balance between oxygen delivery and consumption in the brain, was statistically different between the sham and RIPC groups under the HH condition (sham group: 25.20 ± 3.40% and RIPC group: 18.73 ± 5.85%; *p* < 0.01, Figure 4E); *p* < 0.01, Figure 4F). However, there was no difference in the value for the right forehead between the two groups (data not shown). The main mechanism behind the hypoxia-induced reduction in VO_2_ max is a decrease in SpO_2_ (Wehrlin and Hallen, 2006). Hence, the improvement in aerobic performance plasticity in response to environmental hypoxia in RIPC-intervened participants may arise from changes in the capacity of O_2_ oxygenation.

### 3.3 Effect of RIPC on cardiovascular hemodynamic parameters

**FIGURE 5:**
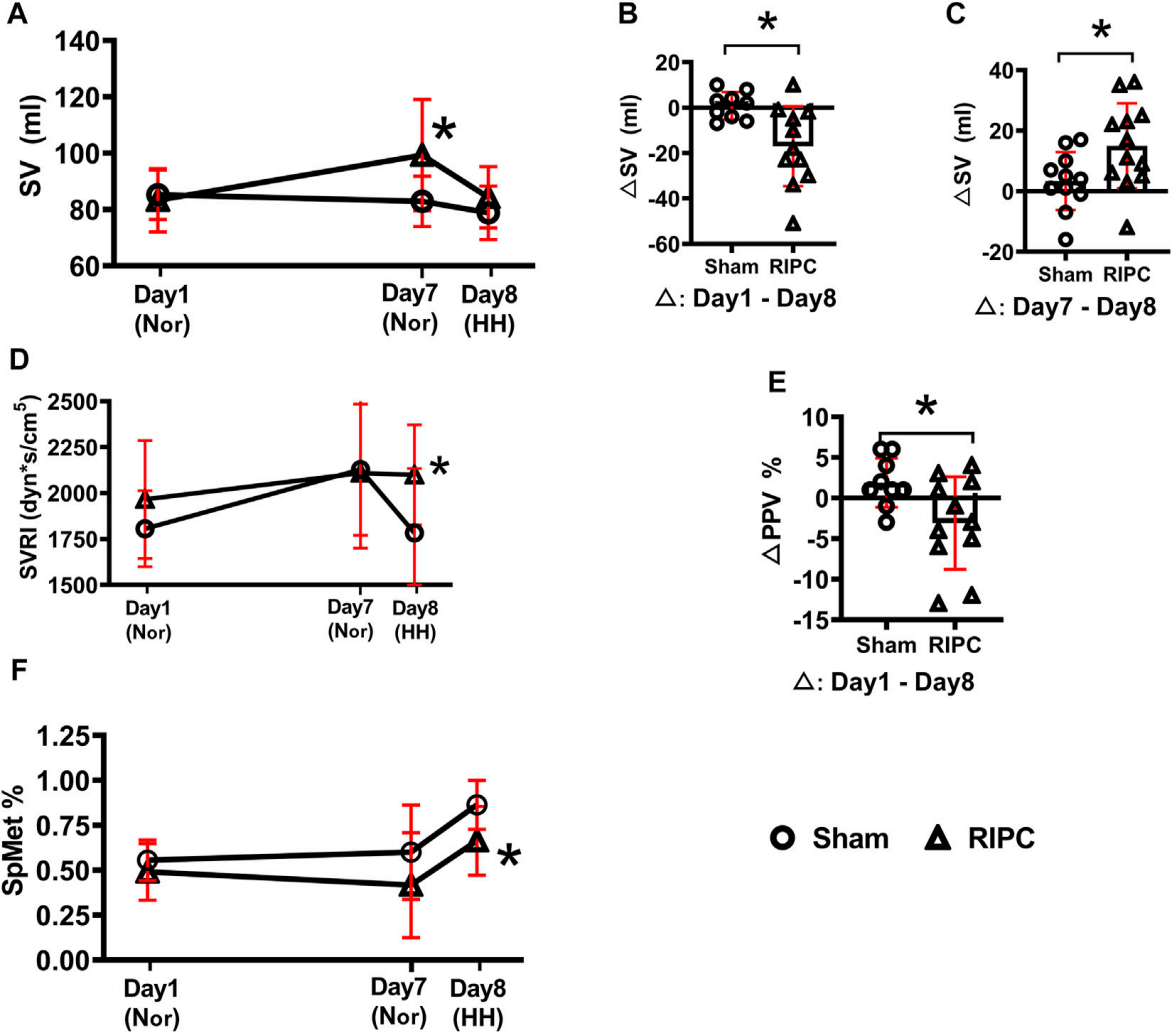
The changes of physiological variables induced by RIPC. **(A)** SV, **(B,C)** △SV, **(D)** SVRI**, (E)** △PPV, and **(F)** SpMet and from normoxic to HH condition in each group. *p < 0.05 vs. sham group. Values are presented as mean ± standard deviation. Nor: normoxic, HH: hypobaric hypoxia, RIPC: remote ischemic preconditioning, SV: stroke volume, SVRI: systemic vascular resistance index, PPV: pulse pressure variation, SpMet: methemoglobin saturation.

To investigate the underlying mechanisms of RIPC intervention in HH-induced aerobic performance variation, several critical physiological variables for maintaining fitness were recorded (Table 2 and Figure 5). A significant difference was found in SV data in the normoxic condition after 8 consecutive days of RIPC between the two groups. Moreover, in the RIPC group, we found that the change in SV decreased significantly between the 1^st^ and 8^th^ day (∆1), and increased significantly between the 7^th^ and 8^th^ day (∆2) (*p* < 0.05, Table 2 and Figures 5B,C). The results showed that SVRI increased significantly compared to sham after 8 consecutive days of RIPC intervention in the HH condition (*p* < 0.05, Table 2 and Figure 5D). Meanwhile, the change in PPV% between the 1^st^ and 8^th^ day (∆1) in the RIPC- intervened participants decreased significantly compared to that in the sham group (*p* < 0.05, Table 2 and Figure 5E). Usually, the adverse effect of SpMet is caused by the oxidation of iron in the heme molecule within Hb from the ferrous to the ferric form. The produced globin molecule causes the oxygen dissociative curve to shift to the left, decreasing the ability of Hb to deliver oxygen to the tissues (Hulse et al., 2016). In the present study, participants in the RIPC group showed a significant decrease in the percentage of SpMet on the 8^th^ day after intervention in the HH condition compared to those in the sham group (*p* < 0.05, Table 2; Figure 5F). Collectively, these data suggest that RIPC affects cardiac function by improving SV and SVRI, and SpMet may be responsible for the amelioration of regional oxygenation.

**TABLE 2:** Variation of basal physiological variables.

Characteristics	Normoxic (day 1)			Normoxic (day 7)		Hypobaric Hypoxia (day 8)		∆1: day 1- day 8		∆ 2: day 7 – day 8		
	Sham (*n* = 9)		RIPC (*n* = 11)	Sham (*n* = 12)	RIPC (*n* = 12)	Sham (*n* = 11)	RIPC (*n* = 12)	Sham (*n* = 9)	RIPC (*n* = 11)	Sham (*n* = 11)	RIPC (*n* = 12)	
SBP (mmHg)	115.00 ± 7.98	113.73 ± 9.46		116.50 ± 8.24	122.33 ± 7.69	111.18 ± 12.00	109.33 ± 14.73	2.67 ± 13.66	-8.81 ± 11.4	115.00 ± 7.98		113.73 ± 9.46
DBP(mmHg)	66.00 ± 5.15	68.27 ± 7.93		70.08 ± 8.41	69.67 ± 11.02	67.00 ± 5.78	66.00 ± 11.04	-0.56 ± 6.62	2.09 ± 13.22	66.00 ± 5.15		68.27 ± 7.93
MAP (mmHg)	82.22 ± 5.40	83.64 ± 7.23		85.58 ± 8.22	89.92 ± 9.85	79.64 ± 6.36	80.50 ± 10.90	2.56 ± 9.29	2.73 ± 11.95	82.22 ± 5.40		83.64 ± 7.23
Pulse (bpm)	67.33 ± 7.16	67.55 ± 7.58		62.25 ± 9.96	58.92 ± 4.96	74.82 ± 11.86	69.67 ± 9.28	-6.78 ± 12.63	-1.00 ± 10.21	67.33 ± 7.16		67.55 ± 7.58
PR (bpm)	67.00 ± 5.07	68.00 ± 7.11		63.00 ± 9.72	59.33 ± 6.26	74.18 ± 11.32	69.00 ± 7.32	-6.00 ± 13.61	-0.09 ± 6.36	67.00 ± 5.07		68.00 ± 7.11
SV (ml)	85.22 ± 8.79	83.27 ± 11.24		82.92 ± 8.93	99.33 ± 19.75*	78.82 ± 9.50	84.33 ± 10.88	0.89 ± 6.03	-17.00 ± 17.58*	85.22 ± 8.79		83.27 ± 11.24
SI (ml/m^2^)	48.44 ± 5.05	46.82 ± 5.71		47.25 ± 5.05	54.83 ± 10.22	44.73 ± 3.66	46.33 ± 6.28	3.11 ± 5.13	5.11 ± 19.19	48.44 ± 5.05		46.82 ± 5.71
SVR (dyn*s/cm^5^)	1,054.66 ± 77.73	1,086.27 ± 177.45		1,225.75 ± 182.79	1,172.75 ± 259.46	993.00 ± 172.90	1,012.58 ± 231.63	58.89 ± 198.27	57.55 ± 336.19	1,054.66 ± 77.73		1,086.27 ± 177.45
SVRI (dyn*s/cm^5^)	1805.44 ± 206.40	1965.18 ± 321.47		2,127.50 ± 357.26	2,108.42 ± 408.20	1783.00 ± 350.81	2098.77 ± 272.22*	-269.11 ± 321.63	-132.27 ± 514.16	1805.44 ± 206.40		1965.18 ± 321.47
PPV%	11.11 ± 3.89	9.73 ± 2.97		10.75 ± 5.48	10.83 ± 3.81	8.73 ± 2.90	13.25 ± 6.97	1.89 ± 3.02	-3.09 ± 5.70*	11.11 ± 3.89		9.73 ± 2.97
PSI	87.88 ± 6.90	91.18 ± 5.53		88.00 ± 8.05	88.92 ± 7.24	90.91 ± 5.30	90.50 ± 7.57	-2.56 ± 9.46	0.82 ± 5.15	87.88 ± 6.90		91.18 ± 5.53
CO(l/min)	5.77 ± 0.46	5.80 ± 0.71		5.37 ± 0.83	5.86 ± 1.01	5.91 ± 1.13	5.98 ± 0.73	-0.22 ± 1.39	-0.12 ± 1.15	5.77 ± 0.46		5.80 ± 0.71
CI (l/min/m^2^)	3.32 ± 0.29	3.20 ± 0.33		3.06 ± 0.51	3.23 ± 0.49	3.30 ± 0.56	3.21 ± 0.41	0.00 ± 0.76	0.04 ± 0.57	3.32 ± 0.29		3.20 ± 0.33
CO/CI	1.74 ± 0.09	1.82 ± 0.16		1.76 ± 0.14	1.81 ± 0.13	1.78 ± 0.10	1.83 ± 0.10	—	—	1.74 ± 0.09		1.82 ± 0.16
SVR/SVRI	0.59 ± 0.05	0.55 ± 0.04		0.58 ± 0.04	0.55 ± 0.03	0.56 ± 0.05	0.49 ± 0.11	—	—	0.59 ± 0.05		0.55 ± 0.04
PVI%	24.88 ± 5.40	22.72 ± 5.14		24.18 ± 6.21	24.45 ± 13.93	24.45 ± 4.50	21.00 ± 3.59	-4.09 ± 11.50	1.78 ± 7.56	24.88 ± 5.40		22.72 ± 5.14
PI	8.32 ± 2.75	8.59 ± 1.73		4.43 ± 2.06	4.32 ± 2.81	6.76 ± 2.68	5.46 ± 2.72	1.93 ± 3.62	2.38 ± 4.52	8.32 ± 2.75		8.59 ± 1.73
SpO_2_%	97.33 ± 0.71	97.18 ± 0.75		97.33 ± 0.65′	97.42 ± 1.16	81.73 ± 3.93	80.83 ± 3.33	15.44 ± 4.56	16.45 ± 3.53	97.33 ± 0.71		97.18 ± 0.75
SpMet %	0.56 ± 0.11	0.49 ± 0.15		0.60 ± 0.26	0.41 ± 0.29	0.86 ± 0.14	0.66 ± 0.19**	-0.32 ± 0.15	-0.19 ± 0.26	0.56 ± 0.11		0.49 ± 0.15

Data are presented as mean ± standard deviation. SBP: systolic blood pressure, DBP: diastolic blood pressure, RIPC: remote ischemic preconditioning, MAP: mean arterial pressure, PR: pulse rate, SV: stroke volume, SI: stroke volume index, SVR: systemic vascular resistance, SVRI: systemic vascular resistance index, PPV: pulse pressure variation, PSI: patient state index, CO: cardiac output, CI: cardiac index, PVI: pleth variability index, PI: peripheral perfusion index, SpO_2_: peripheral oxygen saturation, SpMet: methemoglobin saturation. *p < 0.05 vs. sham group. *N* = 9-12.

### 3.4 Effect of RIPC on HRV

HRV is a sensitive and non-invasive tool for evaluating cardiac autonomic adaptation functions, which are the basis for the maintenance of homeostasis and responsible for the varied actions and reactions in physiological systems under different conditions (Vanderlei et al., 2009; Jabbour and Iancu, 2021). Our data showed that HH exposure resulted in significant decrease in mean RR, RMSSD, pNN50, HF power, and SD1, but a significant increase in mean HR, LF power, ApEn, α1 (DFA1), and α2 (DFA2), compared to the normoxic condition, respectively (Table 3). This was consistent with previous reports that acute hypoxic exposure caused a decrease in the total spectral power in HRV (Perini et al., 1996; Sevre et al., 2001). Nevertheless, for the RIPC group, only the mean RR showed a significant decrease in the HH condition compared with that in the normoxic condition, further suggesting that RIPC can positively regulate cardiac status, which impacts regional oxygenation to enhance aerobic performance.

**TABLE 3:** The changes of heart rate variability at rest under the normoxic and HH conditions.

Characteristics	Normoxic (day 1)		4000 m (day 8)	
	Sham (*n* = 12)	RIPC (*n* = 12)	Sham (*n* = 11)	RIPC (*n* = 12)
Mean RR (ms)	1,121.92 ± 169.15	1,018.58 ± 158.56	912.91 ± 171.01***	936.75 ± 75.62*
Mean HR (ms)	54.50 ± 8.40	60.33 ± 9.32	67.91 ± 12.75***	64.5 ± 5.09
SDNN (ms)	51.70 (34.98,76.45)	40.85 (35.43,62.43)	41.70 (25.40,61.20)	43.80 (39.38,59.73)
RMSSD (ms)	57.55 (43.43,111.68)	43.95 (38.10,72.68)	36.20(23.90,62.80*	41.60 (37.55,53.25)
NN50 (beats)	102.00 (51.25,162.75)	73.50 (44.75,151.00)	51.00 (14.00,130.00)	74.50 (49.25,118.00)
pNN50 (%)	41.85 ± 25.65	32.46 ± 21.12	24.72 ± 22.49*	26.80 ± 13.10
RRTI	10.95 (8.15,14.625)	10.81 (9.35,15.23)	10.28 (7.41,13.78)	10.63 (9.22,13.61)
TINN (ms)	269.00 (203.50,375.25)	214.00 (170.50,302.75)	215.00 (123.00,296.00)	226.00 (184.00,267.00)
VLF (ms^2^)	78.50 (55.25,130.75)	76.00 (38.25,113.25)	74.00 (35.00,184.00)	46.00 (20.00,199.50)
LF (ms^2^)	960.00 (368.75,1354.75)	825.00 (596.75,1996.25)	454.00 (287.00,1667.00)	1,223.50 (630.50,2656.75)
HF (ms^2^)	1,027.00 (503.25,2906.75)	563.00 (281.75,1869.00)	428.00 (270.00,1236.00)	574.50 (405.00,907.25)
LFnu	41.58 ± 16.28	56.55 ± 21.31	53.15 ± 15.77*	61.55 ± 22.60
HFnu	58.38 ± 16.30	43.37 ± 21.33	46.66 ± 15.95*	38.38 ± 22.62
TP (ms^2^)	2,285 (1,049.75,4304)	1,554.50 (1,005.50,4007.75)	1,111.00 (641.00,3338.00)	2055.00 (1,549.75,3816.50)
LF/HF ratio	0.81 (0.37,1.00)	1.40 (0.64,2.23)	1.38 (0.78,1.60)	2.43 (0.67,4.70)
SD1	40.75(30.78,79.13)	31.15 (27.00,51.48)	25.60(16.90,44.50) *	29.45 (26.60,37.70)
SD2	60.90 (42.08,76.50)	49.45 (40.33,76.55)	43.90 (28.20,73.80)	53.15 (42.73,75.30)
SD2/SD1	1.30 ± 0.38	1.70 ± 0.69	1.68 ± 0.47*	1.89 ± 0.65
ApEn	1.05 ± 0.07	1.04 ± 0.07	1.13 ± 0.09*	1.09 ± 0.06
SampEn	1.85 ± 0.14	1.80 ± 0.38	1.89 ± 0.22	1.69 ± 0.29
DFA1	0.77 ± 0.24	0.95 ± 0.31	0.97 ± 0.24*	1.02 ± 0.28
DFA2	0.23 ± 0.13	0.22 ± 0.09	0.40 ± 0.11***	0.28 ± 0.12

Data are presented as mean ± standard deviation or medium and first and third quartiles. RIPC: remote ischemic preconditioning, mean RRi, mean RR interval, mean HR: mean heart rate, SDNN: standard deviation of NN intervals, RMSSD: root mean square of successive differences, NN50: number of pairs of successive NNs that differ by more than 50 ms, pNN50: proportion of NN50 divided by the total number of NN (R-R) intervals, RRTI: RR triangular index, TINN: triangular interpolation of NN intervals, VLF: very low-frequency power, LF: low-frequency power, HF: High-frequency power, LFnu: Low-frequency power in normalized unit, TP: total power, SD1: standard deviation of the instantaneous rate variability the rhythm, SD2: standard deviation of the long-term continuous RR intervals, ApEn: approximate entropy, SampEn: sample entropy, DFA1: short-term fluctuations in DFA1: short-term scaling exponent of detrended fluctuation analysis, DFA2: long-term scaling exponent of detrended fluctuation analysis. *p < 0.05 and ***p < 0.001 vs. sham. *N* = 11–12.

### 3.5 Protein MS Data

**FIGURE 6:**
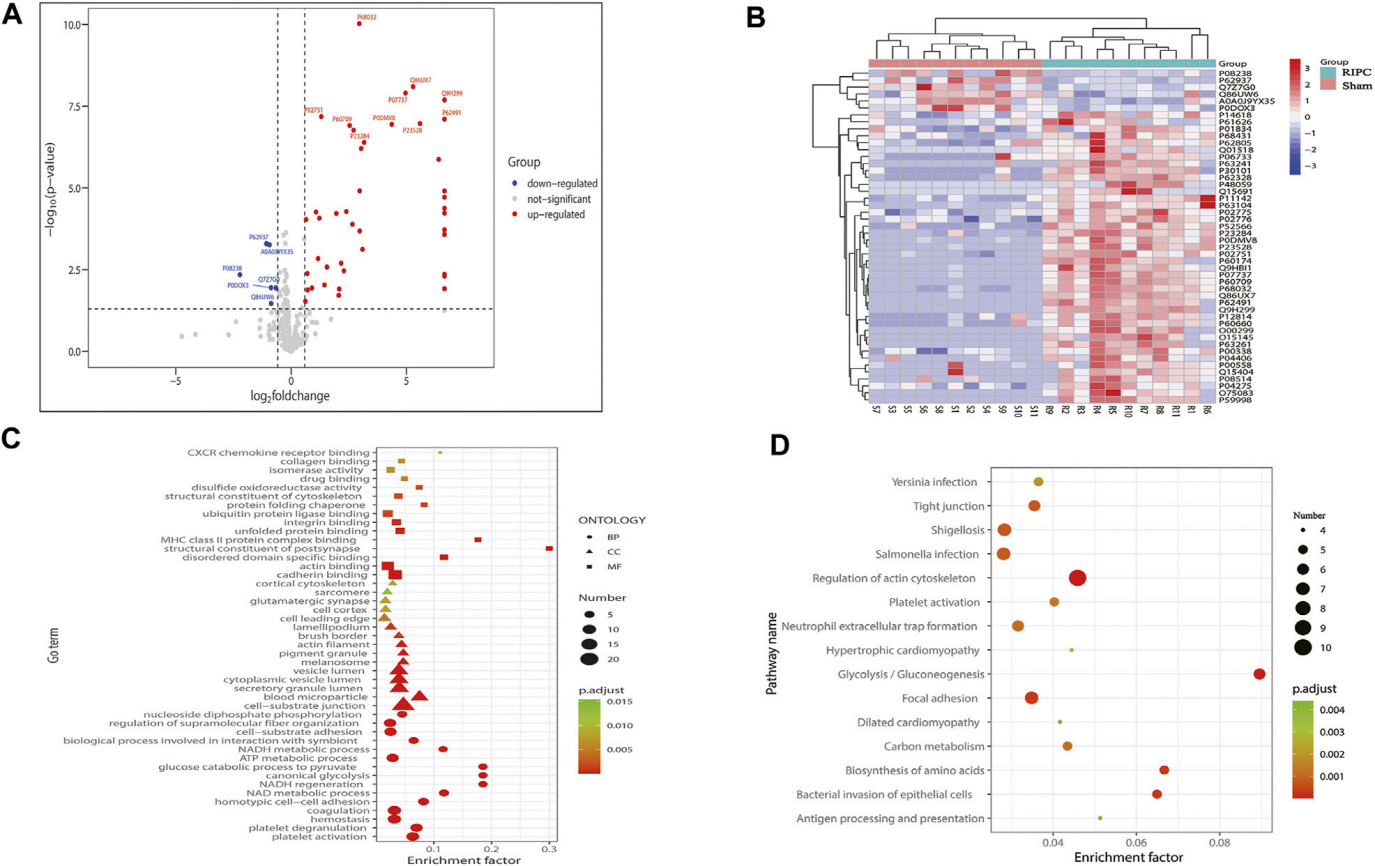
**(A)** Volcano plot of all proteins included in the differential expression analysis. The horizontal axis represents the logarithmic transformation based on 2 of the fold change, and the vertical axis represents the logarithmic transformation based on 10 of the *p*-value. The red dots and blue dots represent proteins with a fold change greater than 1.5 times (upregulation greater than 1.5 times or downregulation less than 0.67 times) and a *p*-value less than 0.05, respectively, and the gray dots represent proteins with no significant difference. The top 10 most significantly upregulated and downregulated proteins are labeled in the plot. **(B)** Hierarchical cluster analysis of the 48 differentially expressed proteins. A tree heat map is used to represent the hierarchical clustering results, with each row representing an individual protein and each column representing an individual sample. Protein expression values are standardized in the heatmap and the relative expression levels of the differentially expressed proteins are shown in different colors, in which the colors closer to red represents a relatively high expression level, and colors closer to blue represents a relatively low expression level. RIPC-(1–11) represent the RIPC group, and sham-(1–11) represent the sham group. **(C)** GO functional enrichment analysis of the 48 differentially expressed proteins. The top 15 most significantly enriched GO terms of each category are showed in the plot. The horizontal axis represents the enrichment factor, and the vertical axis represents the enriched GO function classifications, which are divided into three categories: biological process (BP), molecular function (MF), and cellular component (CC). The shape of the patterning stands for different GO terms. The color of the patterning represents the significance of the enriched GO term. The closer the color is to red, the smaller the *p.* adjust is, and the higher the enrichment significance of the analysis is. The size of the patterning represents the number of differentially expressed proteins involved in a certain GO term. **(D)** KEGG pathway analysis of the 48 differentially expressed proteins. The top 15 most significantly enriched pathways are shown in the plot. The horizontal axis represents the enrichment factor, and the vertical axis represents the significantly enriched KEGG pathways. The color of the dot represents the significance of the enriched KEGG pathway. The closer the color is to red, the smaller the *p.* adjust is, and the higher the enrichment significance of the KEGG pathway is. The size of the dot represents the number of differentially expressed proteins involved in a certain KEGG pathway. RIPC: remote ischemic preconditioning, GO: Gene Oncology, KEGG: Kyoto Encyclopedia of Genes and Genomes.

Protein MS data revealed that 332 proteins with quantitative values at least 50% of the samples within each group, were included in the subsequent differential expression analysis. According to the aforementioned screening threshold standard (Figure 6A), 48 proteins (42 upregulated and six downregulated) were differentially expressed in the RIPC group compared with the sham group (Supplementary Table S1, Figure 6A). A volcano plot was used to visualize the distribution of the differentially expressed proteins. To show the expression profile of the differentially expressed proteins in all samples more intuitively, a hierarchical cluster analysis was conducted (Figure 6B). From the heat map, we can see that the expression profiles of these differentially expressed proteins within groups were very consistent and could effectively separate the two comparison groups, indicating the rationality of screening for differentially expressed proteins. The differentially expressed proteins were significantly clustered in 108 biological process (BP) terms, 27 molecular function (MF) terms, and 21 cellular component (CC) terms (Figure 6C, *p*. adjust<0.05). The top 15 GO terms of each category with the smallest *p*. adjust value and the most significant enrichment were selected for display, and the detailed information of the GO enrichment analysis is shown in Supplementary Table S2. GO annotation analysis revealed that the differentially expressed proteins were primarily distributed in cell-substrate junction, blood microparticle, secretory granule lumen, cytoplasmic vesicle lumen, and vesicle lumen, and were mainly involved in biological processes like platelet activation, platelet degranulation, hemostasis, coagulation, and ATP metabolic process. The enrichment analysis of MF showed that the differentially expressed proteins were primarily enriched in cadherin binding, actin binding, ubiquitin protein ligase binding, etc. We next performed KEGG pathway enrichment analysis to figure out the signal pathways these differentially expressed proteins were involved in. The above differentially expressed proteins were significantly clustered in 27 KEGG pathways (Figure 6D). The top 15 pathways with the smallest *p*. adjust value and the most significant enrichment were selected for display and the detailed information about the enriched KEGG pathways are shown in Supplementary Table S3. Regulation of actin cytoskeleton was the pathway with the smallest *p*. adjust value and was involved in the largest number of differential proteins (*n* = 10). Enrichment factor represents the ratio between the number of differentially expressed proteins involved in a certain KEGG pathway and the total number of the annotated proteins in that pathway. Glycolysis/Gluconeogenesis is the pathway with the highest enrichment factor.

### 3.6 ELISA analysis

**FIGURE 7:**
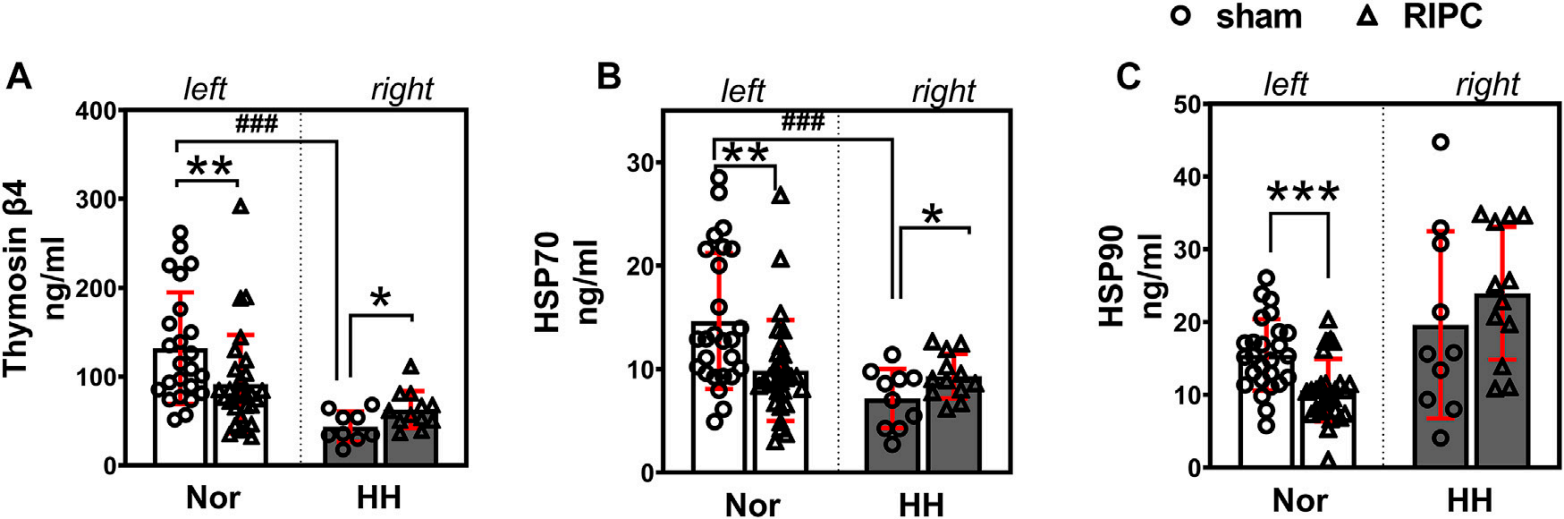
Effect of RIPC on Tβ4, HSP70, and HSP90. **(A)** Tβ4, **(B)** HSP70, and **(C)** HSP90 levels in participants’ plasma after 8 days of RIPC before or after HH exposure. Results are expressed as mean ± standard deviation. ^*^ *p* < 0.05, ^**^ *p* < 0.01,^***^ *p* < 0.001 vs. sham group; ^###^ *p <* 0.001 vs. normoxic. Nor: normoxic, HH: hypobaric hypoxia, RIPC: remote ischemic preconditioning.

To validate the results of the proteomic analysis, three key proteins—Tβ4, HSP70, and HSP90—which have been reported to be involved in the regulation of cardiac function (Bock-Marquette et al., 2004; Smart et al., 2011; Chakafana et al., 2021; Chen et al., 2021), exercise performance (Davison and Brown, 2013; Gonzalez-Franquesa et al., 2021; Samadian et al., 2021) and high-altitude acclimatization (Jain et al., 2013) were chosen for further study by using ELISA kits. In addition, the expression of Tβ4, HSP70, and HSP90 were detected in another independent validation cohort after 8 consecutive days of RIPC intervention without HH exposure. The data showed that plasma Tβ4 (normoxic: 131.87 ± 62.86 ng ml^−1^ and hypobaric hypoxia: 43.44 ± 17.17 ng ml^−1^; *p <* 0.001, Figure 7A) and HSP70 (normoxic: 14.65 ± 6.57 ng ml^−1^ and hypobaric hypoxia: 7.17 ± 2.86 ng ml^−1^; *p <* 0.001, Figure 7B) levels decreased after acute HH exposure due to acute HH injury. Surprisingly, the Tβ4 (sham group: 131.87 ± 62.86 ng ml^−1^ and RIPC group: 91.53 ± 55.30 ng ml^−1^; *p <* 0.01, Figure 7A, left), HSP70(sham group: 14.65 ± 6.57 ng ml^−1^ and RIPC group: 9.87 ± 4.89 ng ml^−1^; *p <* 0.01, Figure 7B, left), and HSP90 (sham group: 15.48 ± 4.91 ng ml^−1^ and RIPC group: 10.62 ± 4.30 ng ml^−1^; *p <* 0.001, Figure 7C, left) levels decreased tremendously before 4,000 m HH exposure after 8 consecutive days of RIPC, compared with those in sham participants. Notably, after HH exposure, Tβ4 (sham group: 43.44 ± 17.17 ng ml^−1^ and RIPC group: 62.68 ± 20.85 ng ml^−1^; *p <* 0.05, Figure 7A, right) and HSP70 (sham group: 7.17 ± 2.86 ng ml^−1^ and RIPC group: 9.32 ± 2.15 ng ml^−1^; *p* = 0.057, Figure 7B, right) concentrations increased slightly, which was consistent with the MS results. These results further suggest that 8 consecutive days of RIPC modulates the expression of Tβ4, HSP70 and HSP90, and they might be responsible for facilitating regional oxygenation and improving aerobic performance in HH exposure.

## 4 Discussion

We demonstrated that RIPC enhances aerobic performance and improves regional oxygenation during HH exposure. Considerable studies have investigated the role of RIPC in high-altitude diseases (e.g., high-altitude pulmonary edema, acute mountain sickness, and high-altitude cerebral edema) (Berger et al., 2015; Berger et al., 2017; Molano Franco et al., 2019), neurological behavior (Li et al., 2020), and exercise performance in moderate hypoxia (Paradis-Deschenes et al., 2018; da Mota et al., 2019; Wiggins et al., 2019). Our major goal was to gain better insights into the influence of RIPC on aerobic performance and muscle O_2_ delivery under severe HH, a condition at an altitude of approximately 4,000 m or above. Cardiac status was improved after 8 consecutive days of RIPC during severe HH exposure. Meanwhile, Tβ4, HSP70, and HSP90 decreased before HH exposure, whereas Tβ4 and HSP70 were abundantly expressed and upregulated after exposure to HH at 4,000 m. To the best of our knowledge, this is the first study to reveal the underlying mechanism of RIPC in high-altitude acclimatization for aerobic performance.

### 4.1 Maximal oxygen uptake and regional oxygenation

Consistently, our data showed that acute HH exposure resulted in a decrease in VO_2_max and SmO_2_ of the leg during the graded exercise test (Fulco et al., 1998; Wehrlin and Hallen, 2006) accompanied by a reduction in rSO_2_ at rest (Imray et al., 2001; Imray et al., 2005). Interestingly, compared to sham intervention, 8 consecutive days of RIPC alleviated the decrease in VO_2_ max, SmO_2_, and rSO_2_ caused by 4,000 m acute HH exposure. Indeed, several recent investigations have revealed that RIPC can enhance exercise performance (i.e., VO_2_max, time-trial performance, and power output) during cycling, rowing, running, and even handgrip (Jean-St-Michel et al., 2011; Cruz et al., 2015; Cruz et al., 2016; Kilding et al., 2018). Nevertheless, some studies reported that RIPC had no effect on exercise performance (Tocco et al., 2015; Incognito et al., 2016; Marocolo et al., 2016), probably because of the RIPC training protocol (the number and duration of the cycles) and exercise intensity. In particular, the benefits of RIPC on exercise performance are currently being confirmed for high-altitude exposure (Paradis-Deschenes et al., 2018; da Mota et al., 2019). Although the protective mechanisms by which RIPC can enhance exercise performance are not well understood, previous evidence indicated that RIPC could improve muscle blood flow and oxygen delivery (de Groot et al., 2010; Crisafulli et al., 2011). An important possibility is the enhanced vasodilatory response following the occlusion (Mortensen and Saltin, 2014). This reactive hyperemia could improve the distribution of regional skeletal muscle blood flow to match the O_2_ demand with a higher O_2_ extraction (Cruz et al., 2015). During maximum exercise, nearly all the available O_2_ is extracted from the blood, perfusing these active muscles (Shephard, 1977). This indicates that the improvements in the ability of tissues to extract O_2_ from the blood may reduce blood O_2_ in response to HH by storing tissue O_2_, which may account for the subsequent underlying mechanism of the effect of RIPC on exercise performance by increasing tissue O_2,_ but not the SpO_2_. There was no difference between the sham and RIPC groups in SpO_2_ during a tranquil state in the HH condition (as shown in Table 2), indicating that there are disadvantages or other confounding mechanisms involved in the SpO_2_ detection protocol, such as cardiac function. The upregulation of SpMet has been shown to be particularly sensitive to hypoxia of altitude (Gourdin et al., 1975; Arnaud et al., 1979), shifting the oxygen dissociation curve to the left because of the compensation for the affinity of Fe^2+^ and O_2_. This left shift elevates oxygen affinity, resulting in decreased oxygen delivery. Specifically, the reduction of small amounts of SpMet has been identified to promote a right curve after RIPC intervention, which might provide some cardiac protection against hypoxia-induced myocardial damage (Zhang et al., 2007; Francone et al., 2011). As discussed above, the verified increased tissue O_2_ of the leg muscles in the RIPC group might explain the physiological effects of RIPC on improving muscle characteristics to enhance aerobic performance (e.g.,VO_2_max) at high altitude.

### 4.2 Cardiovascular hemodynamic and heart rate variability response

The adaptation of VO_2_max in response to exercise is generally due to variations in cardiovascular hemodynamic and heart rate variability response (Bassett and Howley, 2000; Waldron et al., 2020; Carrasco-Poyatos et al., 2022). The decline in arterial oxygen saturation (SpO_2_) caused by hypobaric hypoxia will trigger a rapid compensatory response of the cardiorespiratory system which aims at supplying adequate oxygen to vital organs. The derived cardiovascular hemodynamic variations include blood pressure, heart rate, CO, SV, plasma volume expansion, etc. (Balasubramanian et al., 1978; Naeije, 2010; Boos et al., 2014; Siebenmann et al., 2017). Previous studies show that pharmacological (iron, dexamethasone) (Saunders et al., 2009; O'Hara et al., 2014) and non-pharmacological therapeutics (intermittent hypoxic, plyometric training) (Andrade et al., 2018; Park et al., 2018) which are beneficial to aerobic performance (e.g.,VO_2_max) for hypoxia acclimatization, are closely associated with enhanced cardiovascular hemodynamic, such as heart rate, CO and SV (Bonetti and Hopkins, 2009; Siebenmann et al., 2011; Holdsworth et al., 2020; Singh et al., 2022). Although the mechanistic pathways underlying RIPC improving aerobic performance remain unclear, studies suggest cardiovascular hemodynamic variations link the RIPC stimulus are the most meaningful (Clevidence et al., 2012; Credeur et al., 2019; Paradis-Deschenes et al., 2020). In our study, we did not detect a significant difference in CO between the sham and RIPC groups in the 4,000 m acute HH exposure condition, but the changes in the SV and the SVRI of the RIPC group were prominent. Interestingly, the variation may enhance the cardiopulmonary or arterial baroreflex which lead to increasing plasma volume and O_2_ supply (Floras et al., 2001). These evidence suggested that RIPC intervention may be beneficial to cardiorespiratory system following improved cardiovascular function.

Cardiorespiratory system variations is a complex stress-regulated response for the homeostatic adjustments towards systemic hypoxia, and a central command mechanism and changes in autonomic nervous system (ANS) function played important role in that process (Botek et al., 2015). The hypoxia-induced changes of ANS activity are mainly reflected as an increase in relative sympathetic activity and accompanied by a decrease in parasympathetic activity (Botek et al., 2015). Hypoxia can alter the ANS function during or after exercise. Hypoxia can also lead to an increase in sympathetic activation and a withdrawal of parasympathetic cardiac activity during sub-maximal exercise, and a greater ANS disturbance and a delayed recovery of autonomic balance after exercise (Fornasiero et al., 2019). HRV is a noninvasive method to assess the ANS function, which has been widely adopted in sport and exercise science. Regular moderate exercise training can increase the HRV index, while long-term and continued heavy exercise may lead to fatigue and diminished HRV (Alvarez-Herms et al., 2020). The HRV response may be influenced by the type and intensity of exercise both for aerobic and anaerobic exercises, and it might be an indicative of the adequate intensity of the training (Alvarez-Herms et al., 2020). The pre-exercise autonomic state seems also to be associated with the recovery of HR and HRV indices in the post exercise period, and higher parasympathetic activity at rest corresponded with a faster recovery of HR and HRV indices after exercise (Fornasiero et al., 2018), and higher parasympathetic activity is also suggested to confer higher tolerance for high intensity exercise in elite athletes (Machhada et al., 2017). The effect on autonomic function could be the underlying mechanism of cardioprotection for RIPC, and studies from people shown that some HRV parameters such as the LF/HF ratio, RMSSD, etc. changed after RIPC, which indicated a shift in sympathovagal balance toward greater vagal activity (Chen et al., 2018). Vagal neuro-modulation may do good to reduce the impact of pathophysiological contributing factors such as inflammation, oxidative stress, and sympathoexcitation to diseases (Gidron et al., 2018). However, a single bout of RIPC seems not to cause a significant change in HRV, and the accumulated early and late effects by multiple RIPC bouts may amplify the effects of RIPC (Zagidullin et al., 2016). Consistently, our study indicated that RIPC was associated with an attenuated reduction in RMSSD, pNN50, and SD1, and an increase in SD2/SD1, ApEn, SampEn, DFA1, and DFA2, compared with the sham group. This seems consistent with the finding that RIPC may affect cardiac autonomic nervous activity (Lopes et al., 2018; Noronha Osorio et al., 2019), which could contribute to increase VO_2_max following improved cardiovascular function (Grant and Janse van Rensburg, 2013; Elliott et al., 2015) during exercise in high-altitude conditions.

### 4.3 Plasma protein

Previous studies have found that energy metabolism is involved in aerobic performance (De Feo et al., 2003; Gough et al., 2021). Not surprisingly, GO analysis of plasma proteomics revealed that RIPC-treated participants had significant enrichment in BP related to ATP metabolic processes. Additionally, KEGG analysis showed that differentially expressed proteins were significantly enriched in energy metabolism-associated pathways, such as biosynthesis of amino acids and glycolysis/gluconeogenesis, which might explain the underlying mechanism involved in aerobic performance enhancement. This suggests that RIPC intervention led to a change in energy metabolism, enhancing aerobic performance under the HH condition, and further highlights the crucial roles of RIPC in high-altitude acclimatization.

In the present study, we observed that 42 upregulated and six downregulated proteins were differentially expressed in the RIPC group compared to those in the sham group. We found an obvious decrease in Tβ4 levels after RIPC intervention under the normoxic condition, similar to previous reports that Tβ4 is downregulated and implicated in cardiac protection and angiogenesis via cell proliferation, differentiation, and migration due to its role in cytoskeletal reorganization and intracellular signal transduction after hypoxia/reoxygenation treatment (Li et al., 2019). Tβ4 is present in solid tissues and circulating cells, and with its properties of being conserved, water-soluble, and small sized, it can enter the nucleus by diffusion via the nuclear pore (Goldstein et al., 2005). Following acute hypoxic injury, Tβ4 might protect vital organs (heart, brain and kidney) by inducing a form of “recruitment” and/or “accumulation” leading to increases in the levels of Tβ4 in the brain (Zhou et al., 2015) and heart tissues (Bock-Marquette et al., 2004), but a decrease in the plasma. In other words, the highly conserved body machinery protects vital organs from damage under HH exposure. A recent study reports that Tβ4 is an exercise-induced secreted protein that could increase osteoblast proliferation (Gonzalez-Franquesa et al., 2021). Further, Tβ4 might treat cardiovascular diseases and have beneficial effects on exercise performance (Davison and Brown, 2013; Zhu et al., 2016). Consequently, we postulate that Tβ4 is needed or stored in vital organs for self-defense and plastic changes after RIPC intervention, and then that beneficial substance is released to extracellular spaces, even diffusing into the plasma, to increase VO_2_max, following acute HH.

Heat shock proteins (HSP) are “stress proteins” that are induced by various stressors, such as hypoxia and physical exercise. Studies have shown that intracellular 70 kDa heat shock protein (HSP70) suppresses NF-κB inactivation and plays an important anti-inflammatory role (Heck et al., 2017), and extracellular HSP70 binds to toll-like receptors to activate NF-κB and acts as a pro-inflammatory factor (Radons, 2016). Regular physical activity tends to decrease extracellular HSP70 levels and enhance intracellular HSP70 levels, which is correlated with the inhibition of the pro-inflammatory status (Bittencourt and Porto, 2017). Given this, decreased plasma HSP70 levels were observed in both the 8 days of RIPC training (compared to sham in normoxia) and acute HH (compared to sham in HH), which may imply that the pro-inflammatory status was inhibited, similar to the findings of a recent report (Qin et al., 2021). Based on the biphasic pattern of HSP70 (Bharati et al., 2017), stress factors can stimulate intracellular HSP70 expression to maintain homeostasis and enable self-protection by increasing the synthesis and/or releasing less HSP70 (Scholer et al., 2016; Baldissera et al., 2018). Hence, RIPC could increase the intracellular HSP70 store. Intracellular HSP70 protein has a cytoprotective function and may be released into the extracellular environment during stress (Goettems-Fiorin et al., 2016; Heck et al., 2017; Mai et al., 2017). Accordingly, RIPC intervention improved plasma HSP70 in the RIPC group after acute HH, which may result from the diffusion of intracellular HSP70 into the extracellular environment as a chaperokine to elevate immunomodulatory functions by triggering innate and adaptive immunity (Radons, 2016). The decrease in HSP90 expression after RIPC intervention may also be attributed to a biphasic pattern or increased protein turnover during stress. HSP90 is a major transcriptional regulator of HIF-1α, and elevated HSP90 levels impart tolerance to acute HH and exert anti-apoptotic effects on hypoxia-induced cardiomyocyte damage (Wang et al., 2009; Jain et al., 2013). Meanwhile, HSPs are closely associated to VO_2_max by anti-inflammatory cytokines or NK cells to manipulate the immune system (Peake et al., 2005; Horn et al., 2007). Additionally, HSPs have a cardioprotective role in regulating the angiogenic effects against cardiac apoptosis and/or inflammation (Afina et al., 2021; Tukaj et al., 2021) and have the nature to improve VO_2_max (Hinkley et al., 2017).

These evidences, combined with our findings, suggested that Tβ4 and HSPs may contribute to cardioprotection, and as a result, provide benefits to aerobic performance after RIPC intervention during HH exposure. it will be of interest in future research to determine the rates of mRNA and protein synthesis, degradation of Tβ4 and HSPs in vital organs and peripheral blood, both extracellularly and intracellularly, under RIPC intervention strategies for HH exposure.

## 5 Limitations

This study had several limitations. First, the participants had considerable variability in the inter-test intervals due to the long duration of the experiment, and the physical activity, the daily activities, the dietary intake, and hydration of participants during the study were not recorded. Second, RIPC intervention has two phases of protection: the early phase is from minutes (5- to 10- mine) (de Groot et al., 2010; Paradis-Deschenes et al., 2016) and lasts for a few hours (up to 8 h) (Lisboa et al., 2017), and the second late phase is apparent 12–24 h later and lasts 3–4 days (Berger et al., 2015). Our study involved in the first window of condition, we cannot evaluate the influence of timing on the VO_2_ max, SmO_2_ and physiological variables during hypobaric hypoxia exposure. Third, we only examined a small number of Chinese Han males, which may limit the generalizability of our findings to females and other ethnic groups. Last but not least, in order to avoid the damage to the volunteers caused by frequent blood collection, only one blood collection per batch of the experiment, hence, we did not determine the dynamic change mechanism of Tβ4, HSP70 and HSP90, which requires further exploration, potentially in animal experiments.

## 6 Conclusion

Overall, the current study demonstrated that RIPC enhanced aerobic performance while ameliorating regional oxygenation during HH exposure, which may be based on the improvements in cardiac status via Tβ4, HSP70 and HSP90. Our results suggest that RIPC may serve as a new strategy to facilitate and/or accelerate high altitude acclimatization.

## Data Availability

The datasets presented in this study can be found in online repositories. Raw proteomic sequencing and LC-MS/MS data has been deposited to the ProteomeXchange Consortium via the iProX partner repository with the dataset identifier PXD031247.
